# Design and Experiment of Tea Fresh Leaf Sorting Equipment Under Double Negative Pressure Airflow Field Based on CFD‐RSM Coupling Optimization

**DOI:** 10.1002/fsn3.71219

**Published:** 2025-11-14

**Authors:** Xu Zhang, Rongyang Wang

**Affiliations:** ^1^ Huzhou Vocational and Technical College College of Intelligent Manufacturing and Elevator Huzhou China

**Keywords:** finite element simulation, response surface methodology, single factor experiment, sorting of fresh tea leaves

## Abstract

In response to the demand for sorting fresh tea leaves according to the mass gradient, this paper designs a double negative pressure air‐suction sorting equipment; this equipment is composed of a double negative pressure fan unit, a multi‐hole turntable adsorption system, a tea dropping cabin system and a conveyor belt system. Based on Computational Fluid Dynamics (CFD), the airflow field in the equipment was simulated, and the flow field characteristics under different combinations of negative pressure values (Group A: 380/380 Pa, Group B: 320/320 Pa, Group C: 380/320 Pa, Group D: 320/380 Pa) were analyzed. It was found that the velocity gradient distribution of the flow field in Group A was uniform, which was suitable for the sorting of fresh tea leaves at multiple scales. Through single‐factor experiments, the influences of the rotational speed of the multi‐hole turntable (20, 30, 40, 50 r/min), the initial lateral distance of the blade drop from the negative pressure port (30, 40, 50 mm), and the combination of the pressure values of the double negative pressure ports on the separation rate were studied. The results show that: The separation effect is better when the rotational speed is 30 r/min, the distance is 40 mm, and the pressure is 380/380 Pa. The Box–Benhnken response surface method was further adopted to optimize the parameters, and a regression model of the sorting rate (*R* ^2^ = 0.9980) was established. The optimal parameters were determined as follows: the rotational speed of the turntable was 30 r/min, the horizontal falling distance was 35 mm, and both the upper and lower negative pressure ports were 380 Pa. The verification test score selection rate was 77.5%. This study provides a theoretical and experimental basis for the development of efficient sorting equipment for fresh tea leaves. Subsequently, the influence of factors such as the position of the negative pressure port and the vertical distance on the sorting rate can be further explored.

## Graphical abstract

The double negative pressure air suction type tea fresh leaf sorting equipment was designed. Through CFD simulation, single factor test and response surface method optimization, the optimal parameter sorting rate of 77.5% was obtained, which provides a basis for equipment research and development and prospects for subsequent research.

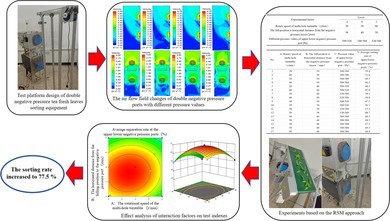

## 1 Introduction

Tea, as an important economic crop in China, its quality directly affects the market value and industrial benefits. The sorting of fresh tea leaves is a crucial link in the tea processing industry chain. It aims to precisely grade based on the morphology of buds and leaves (such as bud heads, one bud with one leaf, one bud with multiple leaves, etc.) and quality differences, in order to meet the processing requirements of different types of tea (Zhang et al. 2022; Jiang et al. 2022; Huang et al. 2023; Liu et al. 2023). Although traditional manual sorting has high precision, it is inefficient and has high labor costs, making it difficult to meet the demands of large‐scale production (Wu 2020; Qiu et al. 2023). Although methods such as mechanical screening (Li et al. 2024; Wang et al. 2020), and color sorting (Cao et al. 2023) have improved efficiency, they have problems such as leaf damage and single sorting standards, especially lacking adaptability to multi‐scale fresh tea leaves. Air flow separation technology has shown broad application prospects in the field of agricultural product grading due to its advantages of non‐contact, low damage and the ability to sort by mass gradient. Its core principle is to utilize the balance between the dynamic force of the air flow and the gravity and inertial force of the materials, and to achieve the separation of materials of different masses by adjusting the air flow and related parameters (Zhang and Zhu 2023; Zhang, Zhu, and Wang 2024; Zhang, Zhu, Yu, et al. 2024; Zhang, Zhu, Yu, and Wang 2024).

CFD provides an efficient tool for gas flow field analysis; it can reveal the velocity and pressure distribution characteristics of the flow field through simulation and guide the optimization of equipment structure (Xiao and Wu 2023; Chang et al. 2023; Li, Zhang, et al. 2023). For example, Bao et al. (2025) explored the flow field structure and the distribution characteristics of dust mass concentration in the new ventilation system through numerical simulation methods and compared them with the traditional ventilation system; Wang et al. (2024) used CFD to explore the influence of inlet air pressure and pipeline length on total pressure loss and pressure distribution in the flow field; Li, Zhu, et al. (2023) conducted simulation experimental research and analysis on the influence of different rotor wind fields on droplet deposition and drift based on the CFD method; Wang et al. (2022) simulated the airflow field of the seed distributor through CFD to study the influence of the seed distributor structure on the uniformity of air pressure. The above‐mentioned scholars all made good use of computational fluid dynamics in their research, conducted preliminary analyses of the experiments, and obtained the optimal experimental conditions. In the research and development of fresh tea leaf sorting equipment, CFD can be used to analyze the uniformity of the flow field under different pressure combinations of double negative pressure fans, providing a basis for determining the optimal negative pressure value. In the early stage, the research group used the CFD simulation method to conduct extensive studies on the design of key components and key factors of tests, and obtained a set of relatively accurate simulation models and model parameters (Zhang, Zhu, and Yu 2024; Zhang, Zhu, Ye, et al. 2024).

In the field of agricultural tea sorting, there are few analysis studies based on the CFD‐RSM coupling method. This method is based on the idea of traditional simulation, experimental verification simulation, and later optimization test, adding the RSM optimization test method; through the coupling of simulation and RSM test, the optimal data results are directly obtained. Chen et al. (2023) used Fluent simulation and Box–Behnken response surface analysis to study the influence of various factors on the success rate of tea collection, and the optimized test result was 26% higher than that before optimization. Chen et al. (2024) analyzed the stress of tea stem clamped by silica with different hardness by CFD, and carried out Box–Behnken test optimization according to the simulation results. After optimization, the success rate of harvesting was increased to 85%. According to the research methods of scholars, based on CFD‐RSM coupling research, the test can be directly optimized and the success rate of the test can be improved.

The Response surface method (RSM), as a multivariate statistical method, can effectively optimize the process parameters under the interaction of multiple factors, and can evaluate the reliability and validity of the model. Through statistical methods such as analysis of variance and goodness of fit test, it can determine the fit degree of the model to the test data and whether the influence of factors on the response variables is significant, thus providing a guarantee for the accuracy and reliability of the test results, it has been widely applied in the parameter optimization of agricultural equipment (Zhao et al. 2025; Wang et al. 2025; Shen et al. 2025; Han et al. 2025; Liu et al. 2024). For example, Chen et al., using the response surface method, with the success rate of harvest as the goal, optimized each factor to obtain the optimal parameters (Chen et al. 2024, 2023); Zeng et al. (2023), taking the success rate of carrot rhizome separation and the damage rate of carrots as response values, used the response surface method to establish regression mathematical models of each factor and the success rate of carrot rhizome separation and the damage rate of carrots.

At present, there are still the following deficiencies in the equipment design and parameter optimization research of the double negative pressure airflow field for multi‐scale sorting of fresh tea leaves: The mechanism of the airflow field distribution under double negative pressure conditions on the adsorption and separation of fresh tea leaves of different qualities is still unclear; The synergistic effect of the combination of the rotational speed of the multi‐hole turntable, the initial position of blade descent and the negative pressure value lacks systematic analysis. The comprehensive sorting efficiency of the existing sorting equipment for multi‐scale leaves such as buds, one bud and one leaf, and one bud and multiple leaves needs to be improved. Based on this, this study designed a double negative pressure air suction type tea fresh leaf sorting equipment. The flow field characteristics under different combinations of negative pressure values were analyzed through CFD simulation. The key parameters were optimized by combining single‐factor experiments and the Box–Benhnken response surface method, aiming to construct an efficient and accurate tea fresh leaf sorting technology system. Provide theoretical and equipment support for the sorting of fresh tea leaves according to the mass gradient.

## 2 Design of Double Negative Pressure Sorting Equipment

**FIGURE 1:**
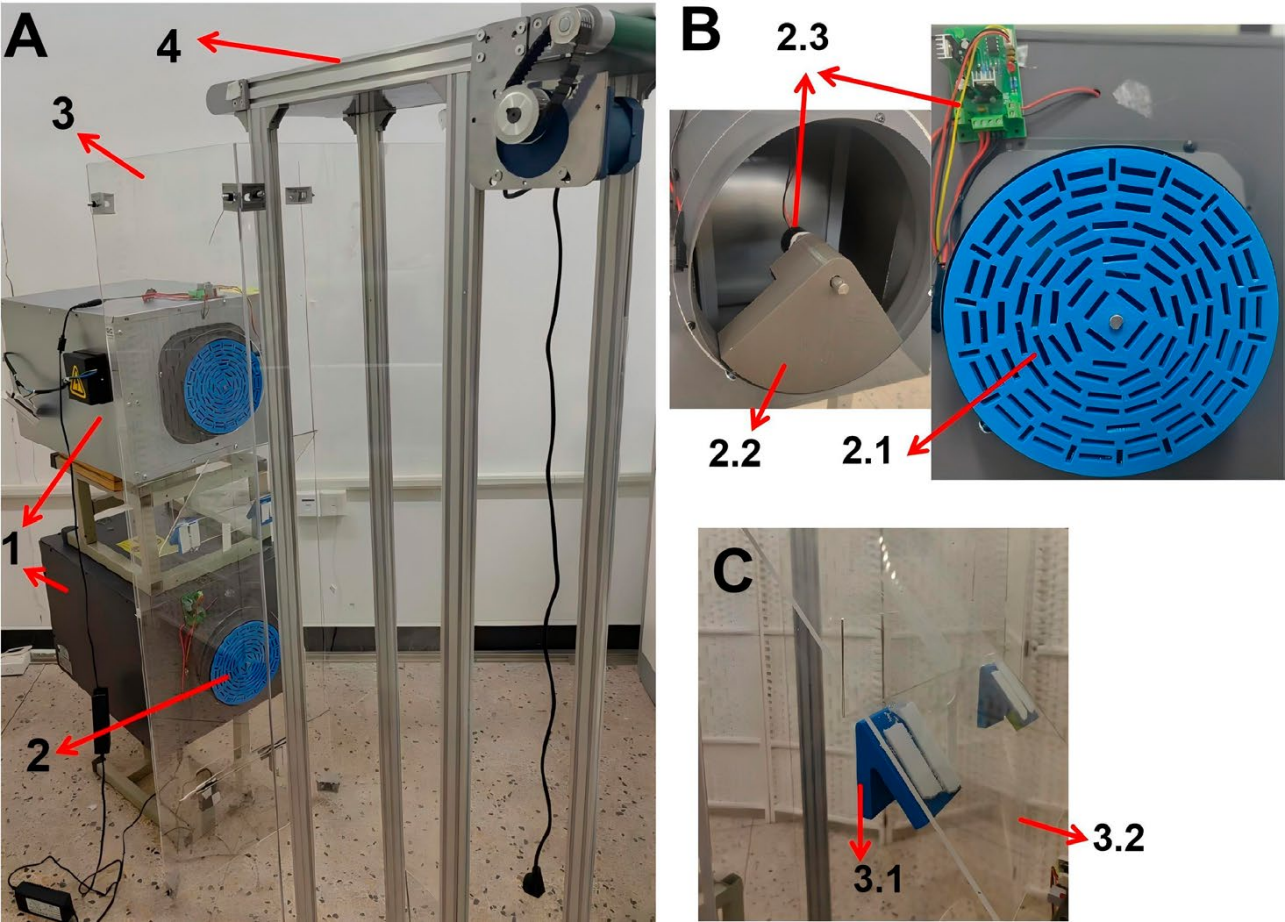
Double negative pressure air suction type tea green leaf sorting equipment. 1: Double negative pressure fan combination; 2: Porous disc adsorption system; 2.1: Multi‐hole turntable; 2.2: Negative pressure baffle; 2.3: Reduction Motor and Control; 3: Tea drop cabin system; 3.1: 40°Angle device; 3.2: Tea slide Board; 4: Conveyor belt system.

The design of the double negative pressure sorting equipment is shown in Figure 1, based on the previous research of single negative pressure (Zhang, Zhu, and Wang 2024; Zhang, Zhu, Yu, et al. 2024; Zhang, Zhu, Yu, and Wang 2024), in order to improve the efficiency of negative pressure sorting; the double negative pressure system is designed. In order to more widely adapt to the family factory, the system is designed with the overall size (including negative pressure fan): length, width and height is 900, 470, 1800 mm. It consists of a combination of double negative pressure fans, a multi‐hole rotating disc adsorption system, a tea drop chamber system and a conveyor belt system. Among them, the multi‐hole rotating disc adsorption system is composed of a reduction motor, a negative pressure baffle and a multi‐hole rotating disc. The tea drop chamber system is composed of a tea drop chamber, a tea slide plate and a 40° angle device. The multi‐hole turntable, negative pressure baffle and 40° angle device of this test platform are all formed by 3D printing technology. The main reason for the 40°design of the falling tea board: (1) tea can slip smoothly; (2) No shielding for negative pressure tuyere; and (3) tilt 40°to facilitate the installation of the tea plate.

The negative pressure isolation plate is fixed at the suction port of the negative pressure fan, and the rotating motor is fixed on the negative pressure isolation plate. The rotating motor shaft drives the multi‐hole rotating plate to rotate. When the negative pressure fan starts, the negative pressure adsorbs the green tea leaves through the holes of the multi‐hole turntable. When the multi‐hole turntable adsorbs the green tea leaves and rotates to the negative pressure isolation plate, the negative pressure is cut off, and the green tea leaves fall to the sliding plate and finally fall into the collection box. Among them, the double negative pressure type refers to the sorting equipment with two negative pressure fans.

When the double negative pressure sorting equipment is in operation, the adsorption force is provided by two negative pressure fans. By adjusting the rotational speed of the motor shaft, the rotational speed of the multi‐hole turntable is controlled. If the rotational speed of the multi‐hole turntable is too fast or too slow, it will lead to a low adsorption rate of tea leaves. After the tea leaves are adsorbed, when the turntable rotates to a non‐negative pressure area, the tea leaves will fall off the turntable, pass through the sliding plate and enter the tea leaves collection box. The suction force of the negative pressure fans used in this study is the same. According to the different characteristics of tea green leaves of the same variety, such as bud heads, one bud and one leaf, one bud and two leaves, one bud and multiple leaves, etc., there are certain differences in their quality. Therefore, the sorting effect is achieved based on the quality gradient difference.

## 3 The Double Suction Sorting Equipment

### 3.1 Negative Pressure Air Flow Field Simulation Analysis of the Flow Field Changes in Control Equation

This chapter mainly studies the movement of the external gas flow field under the influence of the intake and suction flow of the multi‐hole turntable. Now, the gas flow is regarded as a fluid that compensates for compression, viscosity, turbulence and adiabatic effects, satisfying the equations of conservation of mass and conservation of momentum (Fang 2022). The equation of conservation of mass is described as:

(1) $$\frac{\partial }{\partial x_{i}}\left(\rho \mu_{i}\right)=0$$

where $\mu_{i}$ is the velocity component of air in the direction *i*.

The equation of conservation of momentum can be described as:

(2) $$\frac{\partial }{\partial x_{i}}\left(\rho \mu_{i}\mu_{j}\right)=-\frac{\partial H_{j}}{\partial x_{i}}+\frac{\partial \tau_{\mathrm{ij}}}{\partial x_{i}}+F_{i}$$

where $H_{j}$ is static pressure, $\tau_{\mathrm{ij}}$ is stress tensor and $F_{i}$ is volume force.

The airflow adsorbed by the porous turntable outside the negative pressure fan forms a rotating airflow on the outside of the porous turntable as it rotates. Therefore, this belongs to the problem of rotational flow. In this study, the Realizable *k‐ε* turbulence model was adopted in the model calculation. This model is suitable for complex shear flows involving rapid strain, medium vortices and local transitions, and has high accuracy (Rohdin and Moshfegh 2007). The transport equations of *k* and *ε* in the Realizable *k‐ε* model:

(3) $$\frac{\partial \left(\rho \mu_{j}\varepsilon \right)}{\partial x_{j}}=\left(\mu +\frac{\mathrm{\mu t}}{\sigma_{\varepsilon }}\right)\nabla^{2}\varepsilon +C_{1}\mathrm{S\rho \varepsilon}-C_{2}\frac{\rho \varepsilon^{2}}{k+\sqrt{\mathrm{v\varepsilon}}}$$

where *μ* represents the hydrodynamic viscosity, $C_{1}=\max\left[0.43,\frac{\gamma }{\gamma +5}\right]$, $C_{2}=1$, $\sigma_{\varepsilon }=1.2$, ∇ is the Laplace operator.

### 3.2 Model Simplification and Meshing

**FIGURE 2:**
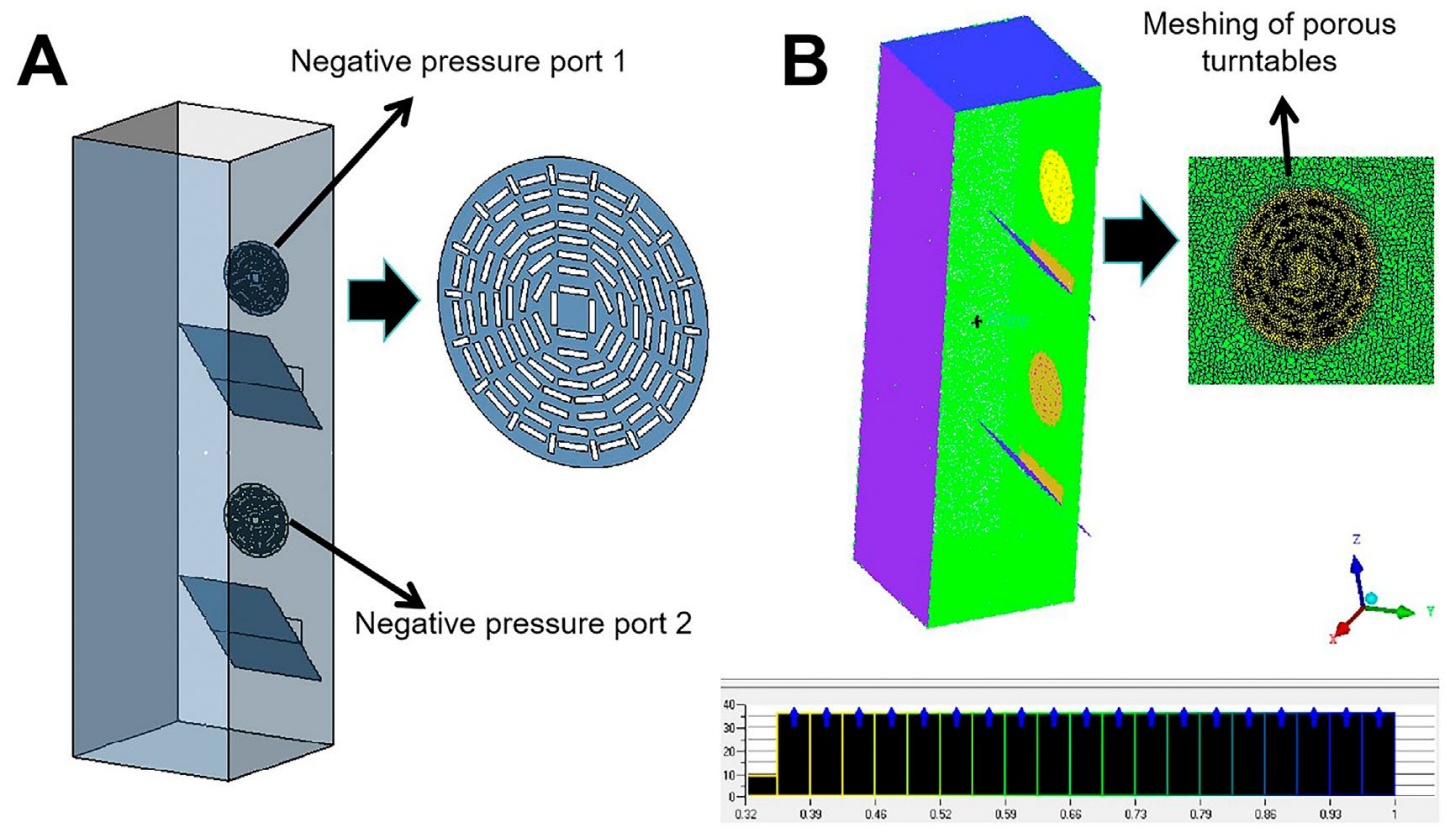
Model simplification and mesh partitioning.

This section mainly discusses the changes in the airflow field of the double negative pressure fans in the tea‐dropping cabin when they have different negative pressure values. The 1:1 model simplification of the double negative pressure sorting equipment was carried out. The simplified model only contains the multi‐hole turntable and the tea dropping chamber, as shown in Figure 2A. The simplified model was meshed by using CFD‐ICEM. The hole surfaces at the multi‐hole turntable and the tea leaf slide plate were filled and set as the pressure outlet “outlet”. The remaining surfaces of the multi‐hole turntable and the tea leaf drop chamber were set as “wall”. The tea leaf slide plate inside the tea drop chamber was set as the internal surface, and the place where the tea leaves dropped was set as the pressure inlet.

Prior to the analysis in this study, a grid independence test has been conducted, and the test results show that the grid size and the grid division strategy have no significant effect on the CFD simulation results. The grid size is divided as follows: In the global element scale factor, the max element size is 10 mm, and the scale factor is 1.2; Separate mesh division was carried out for the hole surface and non‐hole surface of the multi‐hole turntable. The maximum size of the mesh was 2 mm, the growth rate was set to 1.2, the mesh form of the volume mesh was tetra/mixed form, and the mesh method is Robust (Octree); the total of 2.36 million mesh cells were divided (the number of bits was retained to tens of thousands). As shown in Figure 2B, the minimum orthogonal mass of the grid is 0.319, the maximum orthogonal mass is 0.998, the average mass is 0.899; there is no negative grid, and simulation calculation can be carried out.

### 3.3 Analysis of Changes in the Double Negative Pressure Airflow Field

**FIGURE 3:**
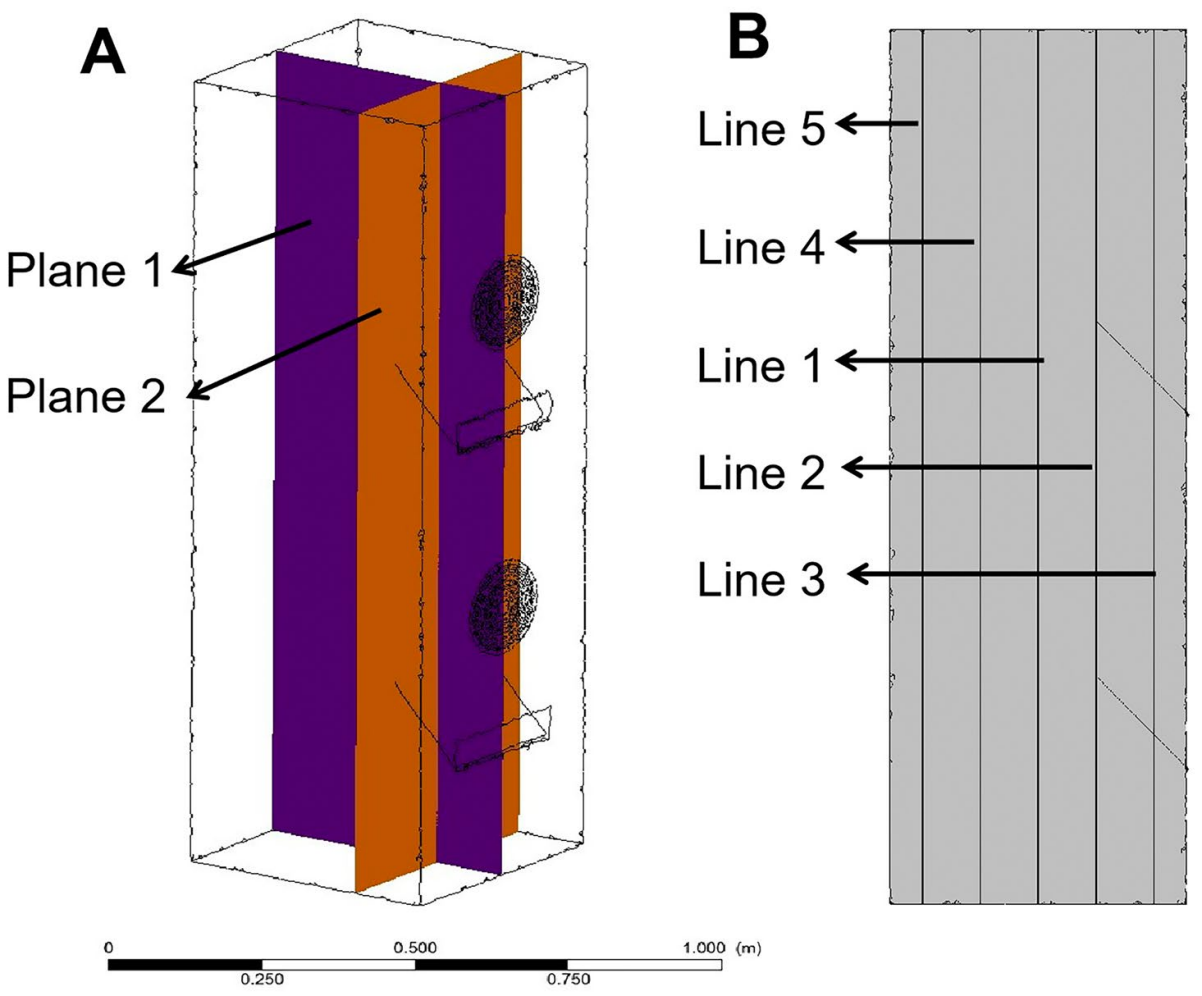
The planes and lines established in the analysis of the airflow field.

Double negative pressure air flow field changes for the selected for testing the negative pressure fan pressure double bar adjustable, negative pressure value of the two gear 380 Pa and 320 Pa, respectively, to obtain the best combination negative pressure value, this section will, under the negative pressure fan on grouping, combinations of different negative pressure values of negative pressure air flow field analysis, among them, Group A has a negative pressure value of 380 Pa for the upper fan and 380 Pa for the lower fan. Group B has a negative pressure value of 320 Pa for the upper fan and 320 Pa for the lower fan. Group C has a negative pressure value of 380 Pa for the upper fan and 320 Pa for the lower fan. In Group D, the negative pressure value of the upper fan is 320 Pa and that of the lower fan is 380 Pa. This section will be based on the Plane (Plane 1) perpendicular to the multi‐hole turntable and the Plane (Plane 2) parallel to the multi‐hole turntable, where Plane 2 is 15 cm away from the multi‐hole turntable, as shown in Figure 3A. Five straight lines were selected on the plane, and 100 data points were chosen on each line for velocity change analysis, as shown in Figure 3B.

#### 3.3.1 Cloud Map Analysis and Data Line Analysis at Plane 1

**FIGURE 4:**
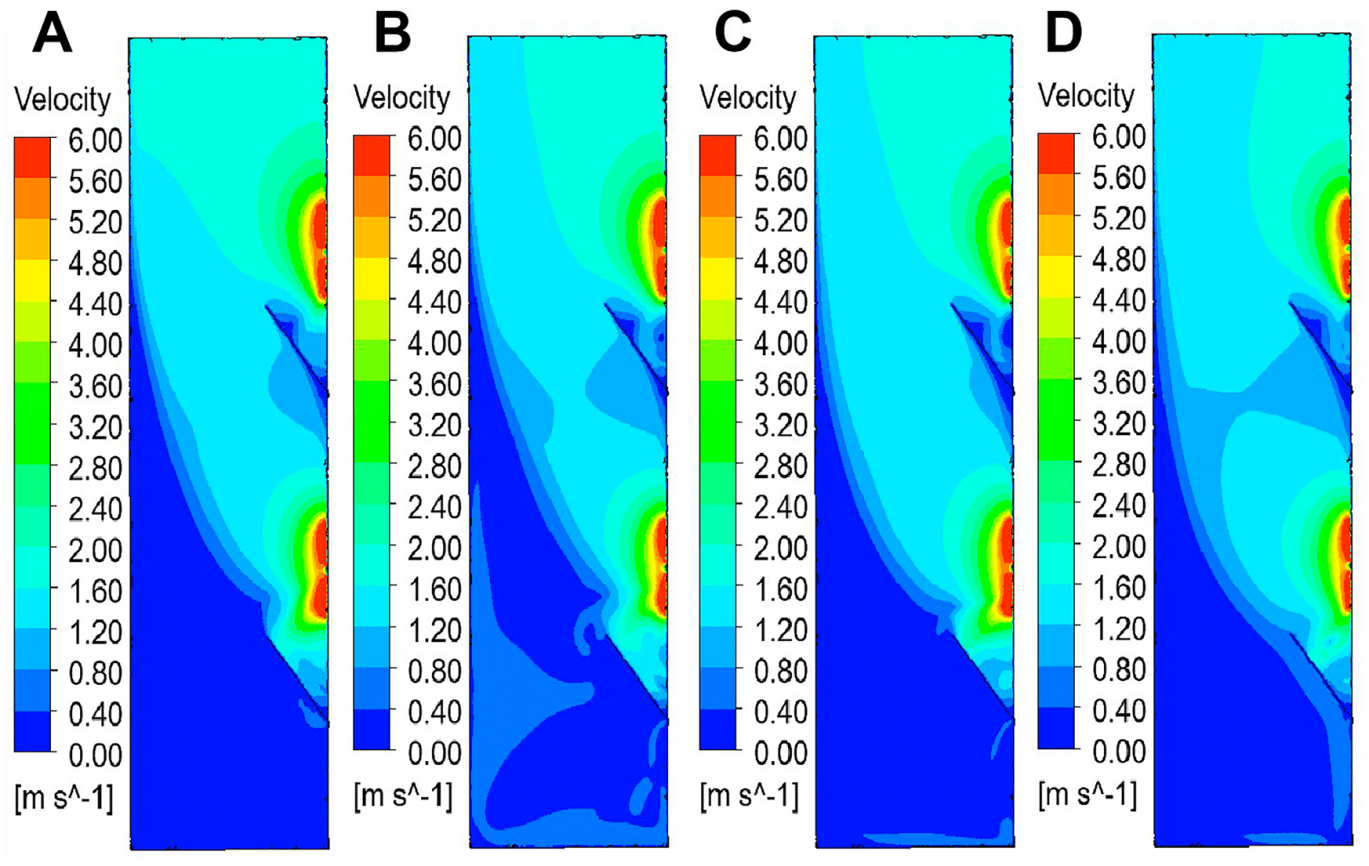
The changes of the airflow field cloud maps of Groups A–D on Plane 1.

The plane perpendicular to the middle position of the multi‐hole turntable was selected for cloud map analysis. The four groups of cloud maps A, B, C, and D all adopted the same velocity scale (0.00–6.00 m/s) and color level mapping rules, which were convenient for directly comparing the velocity distribution characteristics under different working conditions, as shown in Figure 4.

In Figure 4A, the cloud map at the entrance shows a relatively small change in wind speed gradient, meaning that the velocity at the negative pressure outlet 2 cannot affect the entrance. The negative pressure wind speed at the entrance is mainly determined by the negative pressure outlet 1; the bottom and the blue area indicate that the negative pressure wind speed has a relatively small and stable impact, Figure 4A generates a velocity gradient inside the tea drop chamber, and this form of velocity gradient is suitable for sorting multi‐scale tea green leaves with large quality differences. In Figure 4B, although there is a velocity gradient at the wind speed inlet, the proportion of the low‐speed area at the inlet is relatively small. At the tea leaf slide plate at the negative pressure inlet 1, the velocities on both sides are relatively small, resulting in a larger velocity gradient area. A more obvious low‐speed vortex structure appears at the lower part of the middle (the extension of the blue area is more complex), indicating that there is flow separation or vortex phenomenon at this location. The velocity gradient in Figure 4C is similar to that in Figure 4B. The difference lies in that the area of the velocity region changing from the negative pressure port 2 in Figure 4C is larger than that in Figure 4B; moreover, there is no obvious velocity gradient at the tea slide plate of the negative pressure port 1, meanwhile, compared with Figure 4B, there is no obvious low‐speed vortex structure at the middle‐lower position. In Figure 4D, a velocity gradient is also generated at the entrance, The high‐speed and low‐speed areas of the velocity gradient at this location are relatively evenly distributed; the area with low wind speed is far from the negative pressure outlet, while the area with high wind speed is close to the negative pressure outlet. At the tea leaf slide plate below the negative pressure outlet 1, a velocity gradient also appears, and the lower wind speed area cuts off the larger velocity area.

In summary, Figure 4A meets the requirements of the multi‐scale negative pressure sorting velocity gradient. That is, when the multi‐scale tea leaves fall, the heavier ones are not adsorbed by the negative pressure port 1 and fall with the airflow, but are adsorbed by the negative pressure port 2. In Figure 4B–D, velocity gradients all appear at the velocity inlet. The low‐speed area is far from the negative pressure port. During negative pressure sorting, it needs to be determined based on the lateral distance from the initial fall to the negative pressure port. Compared with Figure 4A, there are more variables to be judged. Therefore, this study will conduct experimental analysis in the form of the flow field in Figure 4A.

**FIGURE 5:**
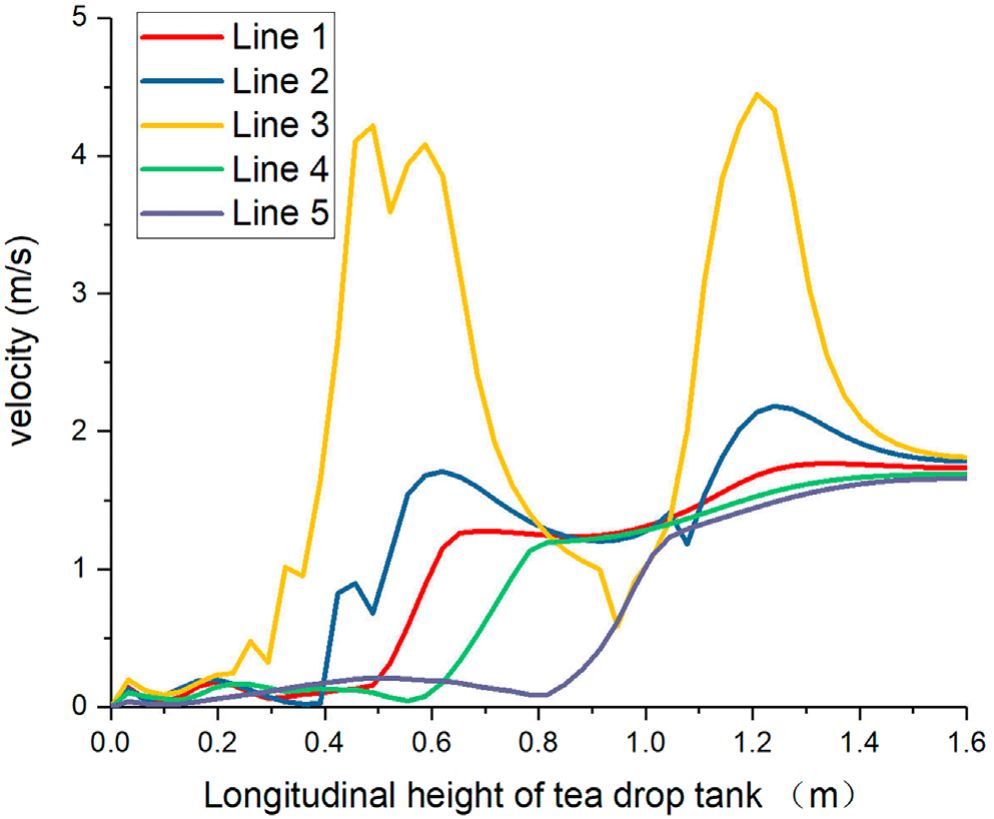
The velocity variations of each line on Plane 1 in Group A.

Based on the above analysis, the cloud map plane of Group A in Figure 4 is now selected for data analysis on regional straight lines, that is, the velocity change values of the five straight lines in Figure 3B. Among them, the horizontal coordinate represents the height of the tea drop cabin and the distance from the bottom to the entrance. The vertical coordinate represents the change in velocity along the straight line, as shown in Figure 5. At the horizontal coordinate range of 0–0.4 m, the velocities of each line are at a low level (0–1 m/s), and the changes are gentle, indicating that the initial flow in the bottom area of the tea drop can is stable and not strongly disturbed. At 0.4–0.8 m, the velocity changes are significantly differentiated. Among them, the velocity of Line 3 suddenly rises to approximately 4 m/s. The reason is that this area is a negative pressure port 2, which is an airflow acceleration structure. Fluctuations occur in Line 2 and Line 5, reflecting that the airflow in this area is affected by the structure and generates a velocity gradient. In the range of 0.8–1.2 m, the velocities of each line tend to stabilize, the fluctuations decrease, and the system flow gradually adjusts to a relatively stable state. After 1.2 m, Line 3 climbed rapidly again at a speed of over 4 m per second. The reason is that this area is a negative pressure port 1, and the relatively high wind speed adsorbs the tea leaves. The speed changes of the remaining lines are relatively slow and remain at about 2 m/s.

The overall velocity distribution shows a phased characteristic of fluctuating changes with the increase of longitudinal height. This design provides support for the precise sorting of tea leaves according to their shape, weight and other characteristics through differentiated airflow dynamic conditions, and also reflects the regulatory role of structural design on airflow movement, providing data references for optimizing sorting efficiency and improving equipment structure.

#### 3.3.2 Cloud Map Analysis and Data Line Analysis at Plan 2

**FIGURE 6:**
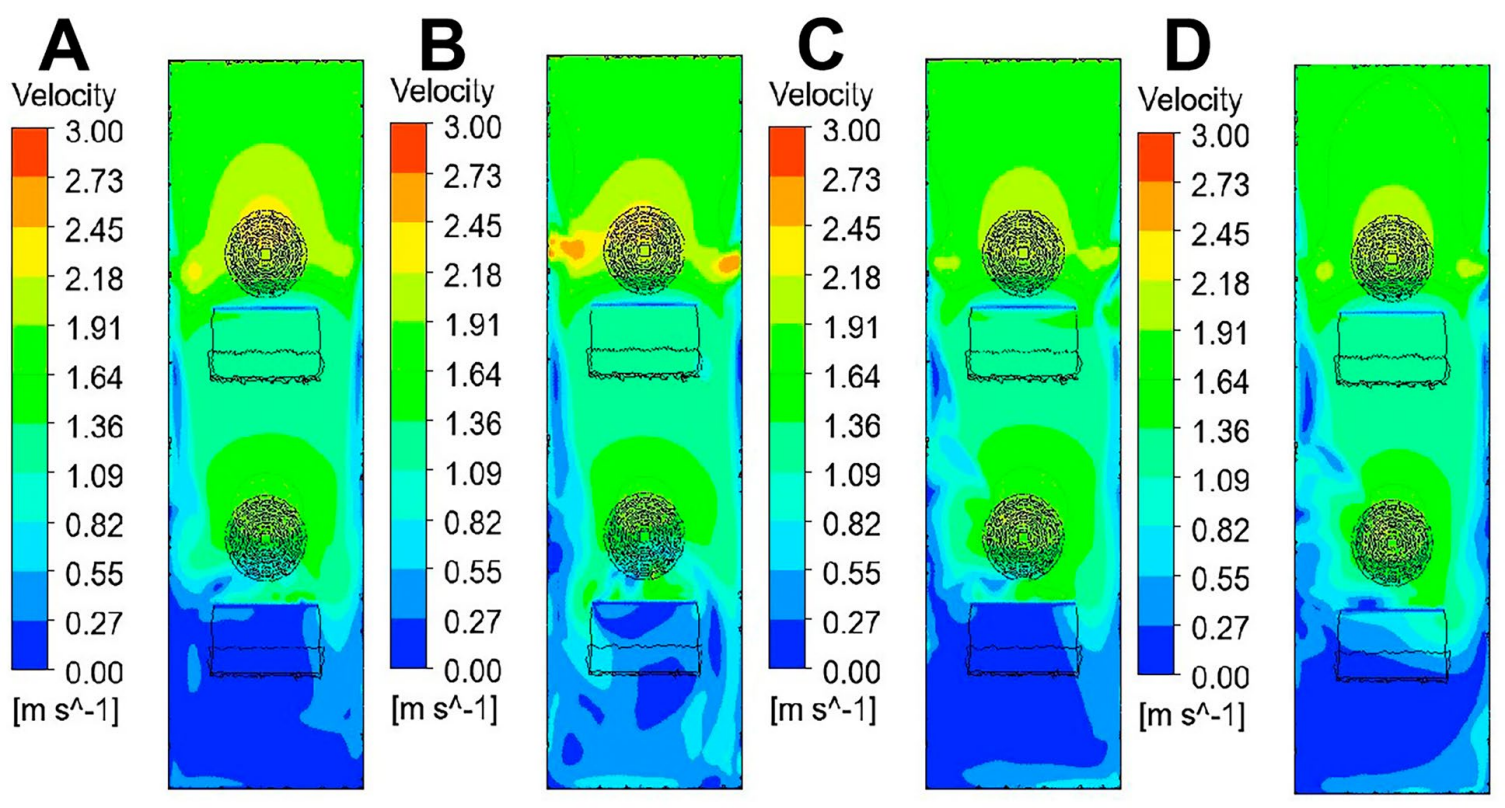
The changes of the airflow field cloud maps of Groups A–D on Plane 2.

The plane perpendicular to the middle position of the multi‐hole turntable was selected for cloud map analysis. The four groups of cloud maps A, B, C, and D all adopted the same velocity scale (0.00–3.00 m/s) and color level mapping rules, which were convenient for direct comparison of the velocity distribution characteristics under different working conditions, as shown in Figure 6A.

Through the cloud map, the variation characteristics of the overall airflow field are analyzed: In Figure 6A, the airflow field is relatively uniform, and the transition between the high‐velocity area and the low‐velocity area is relatively smooth, showing a relatively stable airflow distribution pattern; Compared with Figure 6A, the non‐uniformity of the airflow field is more obvious. Figure 6B shows a concentrated area of high flow velocity locally; Figure 6C shows that the shape and position of the high‐velocity area have changed; A large area of high flow velocity appears in the upper part of Figure 6D, and the overall pattern is significantly different. The airflow variation near the double negative pressure air outlets is analyzed: As shown in Figure 6A, the flow velocity near the air outlets changes in an orderly manner. The flow velocity gradually adjusts from the air outlets outward, which is conducive to forming regular airflow guidance and ensuring the stability of the airflow inside the cabin. As shown in Figure 6B, the flow velocity near the air outlet fluctuates greatly, and there are local high‐speed areas, which may cause airflow disorder and affect the pressure stability inside the negative pressure chamber. As shown in Figure 6C, the flow velocity gradient around the air outlet is different from that in Figure 6A. This difference may interfere with the adsorption path of the airflow to the blades and reduce the adsorption capacity. As shown in Figure 6D, the diffusion pattern of the airflow flowing out of the air outlet is different from that in Figure 6A. A large area with high flow velocity will disrupt the airflow inside the cabin, which is not conducive to the adsorption of the blades. The above analysis indicates that the variation of the airflow field in the plane of Figure 6A conforms to the requirements of multi‐scale fresh tea leaf sorting in this experiment.

## 4 Experiment and Optimization of Influencing Factors of Double Negative Pressure Separation

### 4.1 Single Factor Experiment

The tea sorting rate is an indicator that measures the proportion of tea leaves effectively separated according to specific quality standards (such as shape, size, etc.) during the tea sorting process, reflecting the accuracy and effectiveness of the tea sorting equipment. Its expression form:

(4) $$\varphi =\frac{A}{W}\times 100\%$$

where *A* is the actual quantity (or quality) of tea that has been sorted and meets the target standards; *W* is the total quantity (or quality) of the sorted tea leaves.

According to the previous experiments of the research group (Zhang et al. 2025), the rotational speed of the multi‐hole turntable, the initial lateral distance from the blade drop to the negative pressure port, and the negative pressure value—these three factors directly affect the separation rate of the negative pressure sorting equipment. This section will conduct single‐factor tests on the above three factors in the double negative pressure sorting test.

#### 4.1.1 Single‐Factor Test of the Rotational Speed of the Double Negative Pressure Multi‐Hole Turntable

To explore the influence of the working speed of the multi‐hole turntable on the adsorption performance of fresh tea leaves in the double negative pressure sorting equipment, a single‐factor bench test was carried out with the working speed of the multi‐hole turntable as the test factor. Among them, the upper negative pressure port was 380 Pa, the lower negative pressure air port was 380 Pa, the initial lateral distance from the leaf drop to the upper negative pressure port was 50 mm, and the vertical distance from the upper negative pressure port was 420 mm. Through the pre‐test, it was found that when the rotational speed of the multi‐hole turntable at the upper and lower negative pressure ports was 30 r/min (the speed of the conveyor belt was moderate and constant), the adsorption effect was better. The rotational speeds of 20, 30, 40, and 50 r/min were selected as the test speeds respectively to determine the rotational speed range of the multi‐hole turntable.

**FIGURE 7:**
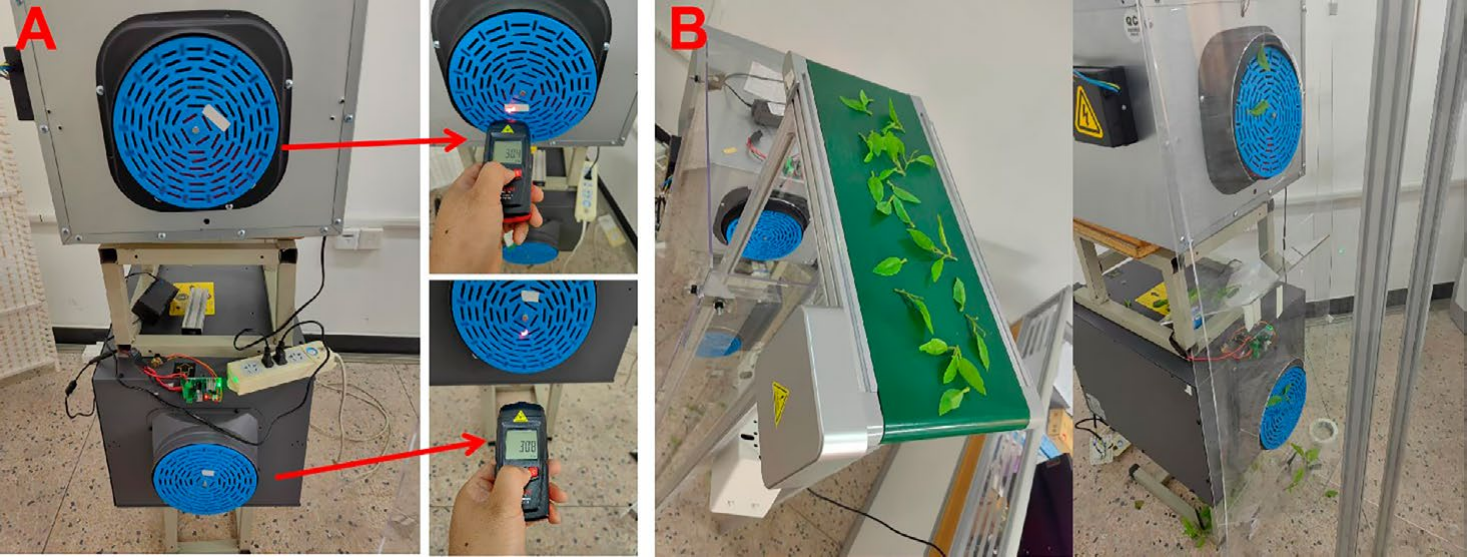
Single‐factor tests of the multi‐hole turntable at different rotational speeds.

A total of 50 tea green leaves were tested at multiple scales, including 15 single leaves, 15 one bud with one leaf, and 20 one bud with two leaves and multiple leaves. A tachometer (Deli Xi Electric Co. Ltd.) was used to measure the speed of the multi‐hole turntable, as shown in Figure 7A. Taking the sorting rate as the test index, the influence of the speed of the porous turntable on the sorting was determined, and the test was repeated three times, as shown in Figure 7B. Through the test, it was found that the speed being fast or slow had a direct impact on the sorting rate. The upper negative pressure port mainly adsorbed single leaves and one bud with one leaf, while the lower negative pressure tuyere mainly adsorbed one bud with two leaves and one bud with many leaves, but the adsorption effect of the lower negative pressure port was generally worse than that of the upper negative pressure port. When the speed of the upper negative pressure port is low, a single leaf or one bud with one leaf will be adsorbed by the lower negative pressure port, and one bud with many leaves will not be adsorbed; the test results are shown in Table 1.

**TABLE 1:** Single‐factor test results of the multi‐hole turntable at different rotational speeds.

Test level factor (r/min)	Test the adsorption rate						Average adsorption rate	
	The first time		The second time		The third time			
	1	2	1	2	1	2	1	2
20	60%	55%	70%	65%	63.3%	65%	64.4%	61.7%
30	66.7%	70%	76.7%	70%	76.7%	75%	73.4%	71.7%
40	60%	65%	53.3%	55%	56.7%	55%	56.7%	58.3%
50	36.7%	40%	43.3%	35%	30%	40%	36.7%	38.3%

*Note:* The upper negative pressure opening is 1, and the total number of fresh tea leaves is 30. The lower negative pressure opening is 2, and the total number of fresh tea leaves is 20.

As the rotational speed of the multi‐hole turntable increases from 20 to 30 r/min, the average separation rate shows an upward trend for both the upper negative pressure port (1) and the lower negative pressure port (2). The upper negative pressure port rose from 64.4% to 73.4%, and the lower negative pressure port rose from 61.7% to 71.7%. When the rotational speed continues to increase from 30 r/min to 40 r/min, the average separation rate begins to decrease. The upper negative pressure port dropped to 56.7%, and the lower negative pressure port dropped to 58.3%. When the rotational speed was further increased to 50 r/min, the average separation rate continued to decrease significantly, dropping to 36.7% at the upper negative pressure port and to 38.3% at the lower negative pressure port. At 20 and 30 r/min, the average separation rate of the upper negative pressure port (with a total of 30 fresh tea leaves) was slightly higher than that of the lower negative pressure port (with a total of 20 fresh tea leaves). When the rotational speed reached 40 and 50 r/min, the average separation rate of the lower negative pressure port decreased slightly less compared to the upper negative pressure port. At each rotational speed, the results of the three tests did not show particularly extreme discretization, indicating that the tests have a certain degree of repeatability.

#### 4.1.2 Single‐Factor Test of the Initial Lateral Distance From the Blade Drop to the Negative Pressure Port

To explore the influence of the distance from the initial falling position of fresh tea leaves to the double negative pressure multi‐hole turntable on the sorting performance, this section takes the distance from the falling position of fresh tea leaves to the multi‐hole turntable as the experimental factor and conducts a single‐factor experiment. In the previous pre‐experiment and the previous research of the research group, it was found that when the negative pressure value and the rotational speed of the multi‐hole turntable are constant, as the initial horizontal distance from the falling position of fresh tea leaves moves away from the negative pressure port, the adsorption rate of fresh tea leaves gradually decreases. When the horizontal distance from the negative pressure port is no more than 40 mm, the adsorption effect of fresh tea leaves is the best. The test levels were set at 30, 40 and 50 mm respectively. The rotational speed of the multi‐hole turntable was 30 r/min. The upper negative pressure port was 380 Pa and the lower negative pressure air port was also 380 Pa.

The test results are shown in Table 2. As the horizontal distance from the falling point of the fresh tea leaves to the negative pressure air outlet increases from 30 mm to 40 mm, the average sorting rate at the upper negative pressure outlet decreases from 78.9% to 73.4%, and the average sorting rate at the lower negative pressure outlet increases from 50% to 71.7%. The reason is that the initial falling distance is relatively short, and the one bud with two leaves and one bud with multiple leaves are adsorbed by the upper negative pressure outlet. When the distance further increased from 40 mm to 50 mm, the average separation rate of the upper negative pressure port dropped significantly to 43.3%, and that of the lower negative pressure port dropped to 45%. Overall, it shows a trend that the farther the horizontal distance from the falling point of the fresh tea leaves to the negative pressure air outlet, the lower the sorting rate.

**TABLE 2:** Single‐factor test results of the horizontal distance from the falling point of fresh tea leaves to the negative pressure air outlet.

Test level factor (mm)	Test the adsorption rate						Average adsorption rate	
	The first time		The second time		The third time			
	1	2	1	2	1	2	1	2
30	76.7%	50%	76.7%	45%	83.3%	55%	78.9%	50%
40	66.7%	70%	76.7%	70%	76.7%	75%	73.4%	71.7%
50	46.7%	50%	40%	45%	43.3%	40%	43.3%	45%

*Note:* The upper negative pressure opening is 1, and the total number of fresh tea leaves is 30. The lower negative pressure opening is 2, and the total number of fresh tea leaves is 20.

#### 4.1.3 Single‐Factor Test With Different Negative Pressure Values at Double Negative Pressure Ports

To explore the influence of different negative pressure values at the double negative pressure ports on the sorting rate of fresh tea leaves, this section conducts a single‐factor experiment with different pressure values at the double negative pressure ports as the experimental factors. Among them, the horizontal distance from the falling point of the fresh tea leaves to the negative pressure port is 40 mm, and the rotational speed of the multi‐hole turntable is 30 r/min, which are fixed values. The experimental levels are set as follows: upper negative pressure port 380 Pa, lower negative pressure port 380 Pa; upper negative pressure port 320 Pa, lower negative pressure port 320 Pa; upper negative pressure port 380 Pa, lower negative pressure port 320 Pa; upper negative pressure port 320 Pa, lower negative pressure port 380 Pa.

The test results are shown in Table 3. Comparing the two groups under the same pressure values, it is found that when the pressure at both the upper and lower negative pressure ports is 380 Pa, the average separation rate at the upper negative pressure port is 78.9%, and that at the lower negative pressure port is 75%. However, when the pressure at both ports is 320 Pa, the average separation rate at the upper negative pressure port drops to 37.8%, and that at the lower negative pressure port drops to 31.7%. It can be seen that under the condition of the same pressure at the upper and lower negative pressure ports, a higher pressure value (380 Pa) corresponds to a higher separation rate. Comparing the two groups under different pressure value combinations, when the pressure at the upper negative pressure port is 380 Pa and that at the lower negative pressure port is 320 Pa, the average separation rate at the upper negative pressure port is 67.8%, and that at the lower negative pressure port is 33.3%. When the pressure at the upper negative pressure port is 320 Pa and that at the lower negative pressure port is 380 Pa, the average separation rate at the upper negative pressure port is 53.3%, and that at the lower negative pressure port is 60%. This indicates that under different pressure value combinations, the separation rate of each negative pressure port is significantly affected by its own pressure, and the negative pressure port with a higher pressure has a relatively higher separation rate. Under the conditions of a fixed multi‐hole turntable speed and a fixed initial drop position of fresh tea leaves, the pressure value of the negative pressure port has a significant impact on the separation rate. When the pressure values are the same, a higher pressure corresponds to a higher separation rate; when the pressure values are different, the separation rate of each negative pressure port is related to its own pressure level.

**TABLE 3:** Single‐factor test results of different pressure values at the double negative pressure ports.

Test level factor (Pa)		Test the adsorption rate						Average adsorption rate	
Upper negative pressure port	Lower negative pressure port	The first time		The second time		The third time			
		1	2	1	2	1	2	1	2
380	380	76.7%	70%	76.7%	75%	83.3%	80%	78.9%	75%
320	320	36.7%	30%	36.7%	35%	40%	30%	37.8%	31.7%
380	320	66.7%	35%	70%	30%	66.7%	35%	67.8%	33.3%
320	380	53.3%	65%	56.7%	60%	50%	55%	53.3%	60%

*Note:* The upper negative pressure opening is 1, and the total number of fresh tea leaves is 30. The lower negative pressure opening is 2, and the total number of fresh tea leaves is 20.

### 4.2 Response Surface Method Test and Analysis

#### 4.2.1 Determination of Response Surface Factors

Based on the results of the single‐factor test, the three levels with better single‐factor test results in each group were selected. The response surface design test was adopted. According to the central combination test design principle of Box–Behnken, the three factors that have a significant impact on the double negative pressure sorting were selected for the test. Each factor was designed with three levels; that is, the three‐factor and three‐level combination test was carried out, as shown in Table 4.

**TABLE 4:** Test design of response surface.

Experimental factor	Level		
	−1	0	1
Rotary speed of multi‐hole turntable (r/min)	20	30	40
The fall position is horizontal distance from the negative pressure tuyere (mm)	30	40	50
Different pressure values of upper/lower negative pressure port (Pa)	380/320	380/380	320/380

#### 4.2.2 Analysis of the Test Results of the Double Negative Pressure Separation Rate Response Surface

The response surface test plan and results of the double negative pressure separation rate are shown in Table 5. The data in Table 5 were analyzed using Design‐Expert 13.0 software to obtain the regression model of the average separation rate of the upper and lower negative pressure ports. The variance analysis is shown in Table 6. Here, *A*, *B*, and *C* represent the coded values of the factors such as the speed of the porous disk, the horizontal distance from the falling position to the negative pressure port, and the different pressure values of the upper and lower negative pressure ports. The regression model of the separation rate *Y* has a *p* value of < 0.001, which indicates that the model is extremely significant. The model's determination coefficient *R* ^2^ is 0.9980, suggesting that the model can explain over 99.8% of the variation in the response values, and there is a high correlation between the predicted values and the actual values, with relatively small experimental errors. Predicted *R* ^2^ is 0.9867, Adjusted *R* ^2^ is 0.9954, the difference is less than 0.2, indicating that the fitting effect of the model on the training data is highly consistent with the prediction effect on the new data. There is no overfitting caused by too many dependent variables or the complexity of the model. Meanwhile, the goodness of fit of the model is extremely high, and both are close to 1, indicating that the model has a very strong ability to explain the variation of the response variable. The Adjusted *R* ^2^ is close to *R* ^2^. It is indicated that most of the independent variables introduced in the model are valid and do not contain too many irrelevant or redundant variables. The lack of fit was 0.5963, and the result was not significant. Meanwhile, *R* ^2^, adjusted *R* ^2^, and predicted *R* ^2^ were all relatively high and close, indicating that the model has both good fit and predictive ability, and its results can be trusted.

**TABLE 5:** Test scheme and results.

No.	*A*: Rotary speed of multi‐hole turntable (r/min)	*B*: The fall position is horizontal distance from the negative pressure tuyere (mm)	*C*: Pressure value of upper/lower negative pressure port (Pa)	*Y*: Average sorting rate of upper/lower negative pressure ports (%)
1	20	30	380/380	59.4
2	40	30	380/380	51.6
3	20	50	380/380	49.5
4	40	50	380/380	46.1
5	20	40	380/320	48.5
6	40	40	380/320	45.0
7	20	40	320/380	58.3
8	40	40	320/380	50.0
9	30	30	380/320	47.5
10	30	50	380/320	36.7
11	30	30	320/380	53.3
12	30	50	320/380	48.7
13	30	40	380/380	68.3
14	30	40	380/380	68.3
15	30	40	380/380	67.5
16	30	40	380/380	69.3
17	30	40	380/380	68.8

**TABLE 6:** Analysis of variance of the regression model.

Source	Sum of squares	Degree of freedom	Mean square	*F*	*p*
Model	1664.74	9	184.97	383.30	< 0.0001
*A*—Rotary speed of multi‐hole turntable	66.13	1	66.13	137.03	< 0.0001***
*B*—The falling position is horizontal distance from the negative pressure port	118.58	1	118.58	245.73	< 0.0001***
*C*—Different pressure values of upper/lower negative pressure port	132.85	1	132.85	275.29	< 0.0001***
*AB*	4.84	1	4.84	10.03	0.0158**
*AC*	5.76	1	5.76	11.94	0.0106**
*BC*	9.61	1	9.61	19.91	0.0029***
*A* ^2^	177.07	1	177.07	366.94	< 0.0001***
*B* ^2^	454.10	1	454.10	941.00	< 0.0001***
*C* ^2^	565.10	1	565.10	1171.03	< 0.0001***
Residual	3.38	7	0.4826		
Lack of Fit	1.17	3	0.3900	0.7065	0.5963*
Pure Error	2.21	4	0.5520		
Cor Total	1668.12	16			

*** Extremely significant differences (*p* < 0.01).

** Significant differences (0.01 < *p* < 0.05).

* Non‐significant differences (*p* > 0.05).

The influences of *A*, *B*, *C*, *BC*, *A* ^2^, *B* ^2^ and *C* ^2^ on the separation rate *Y* are extremely significant, and they have significant influences on *AB* and *AC*, indicating that there is an interactive influence among the various variable conditions. Therefore, the test results were processed by using the method of multiple regression fitting, and the regression equations of the effects of the rotational speed of the multi‐hole turntable, the horizontal distance from the falling position to the negative pressure air outlet, and different pressure values at the upper/lower negative pressure ports on the separation rate *Y* were obtained:

(5) $$\begin{array}{cc} Y & =68.52-2.88A-3.85B+4.08C+1.1\mathrm{AB}-1.2\mathrm{AC}\\ & +1.55\mathrm{BC}-6.49A^{2}-10.38B^{2}-11.59C^{2} \end{array}$$

#### 4.2.3 Analysis of the Influence of Interactive Factors on Test Indicators

**FIGURE 8:**
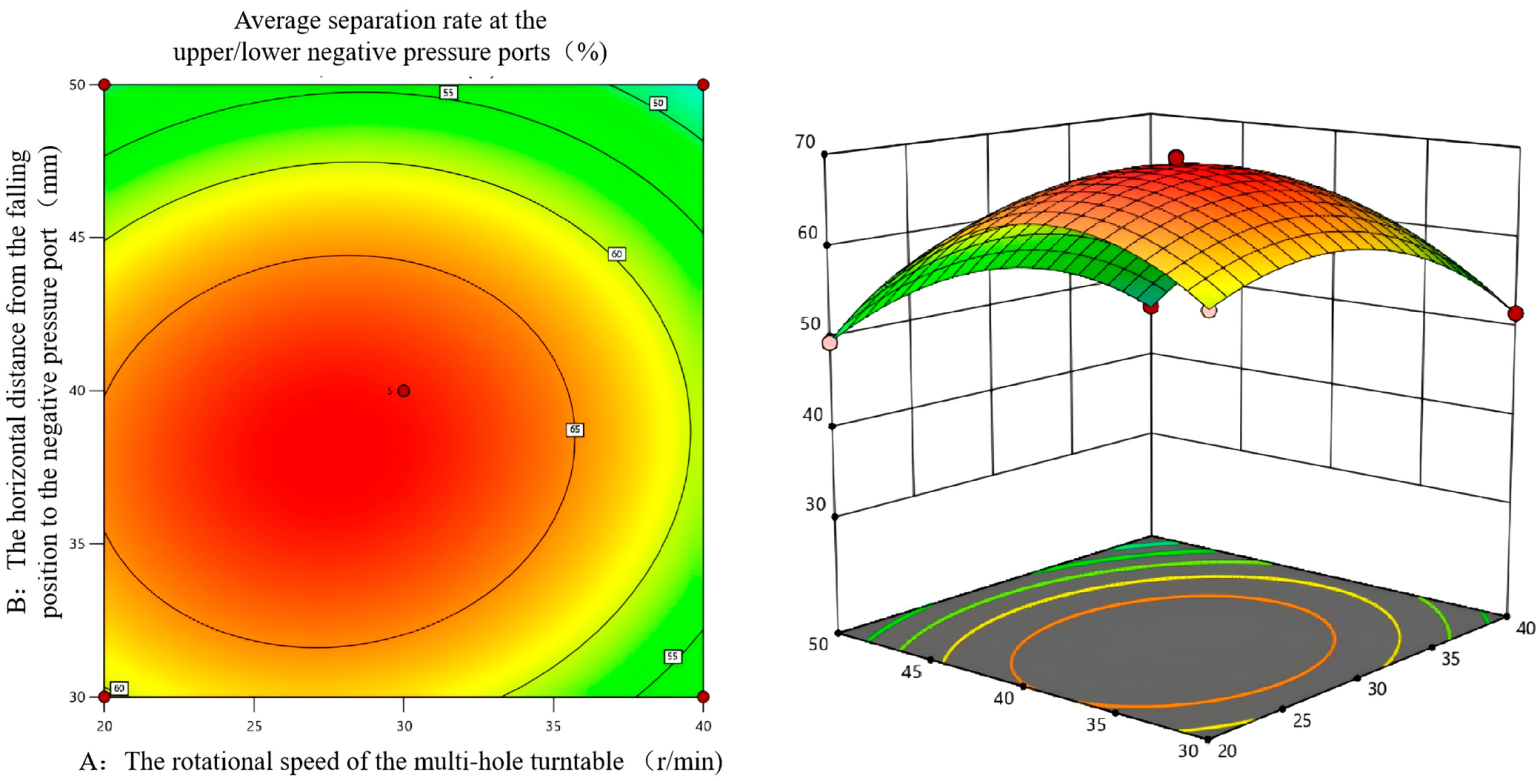
Contour map and response surface map of the influence of the interaction between factors *A* and *B* on the average separation rate under double negative pressure.

**FIGURE 9:**
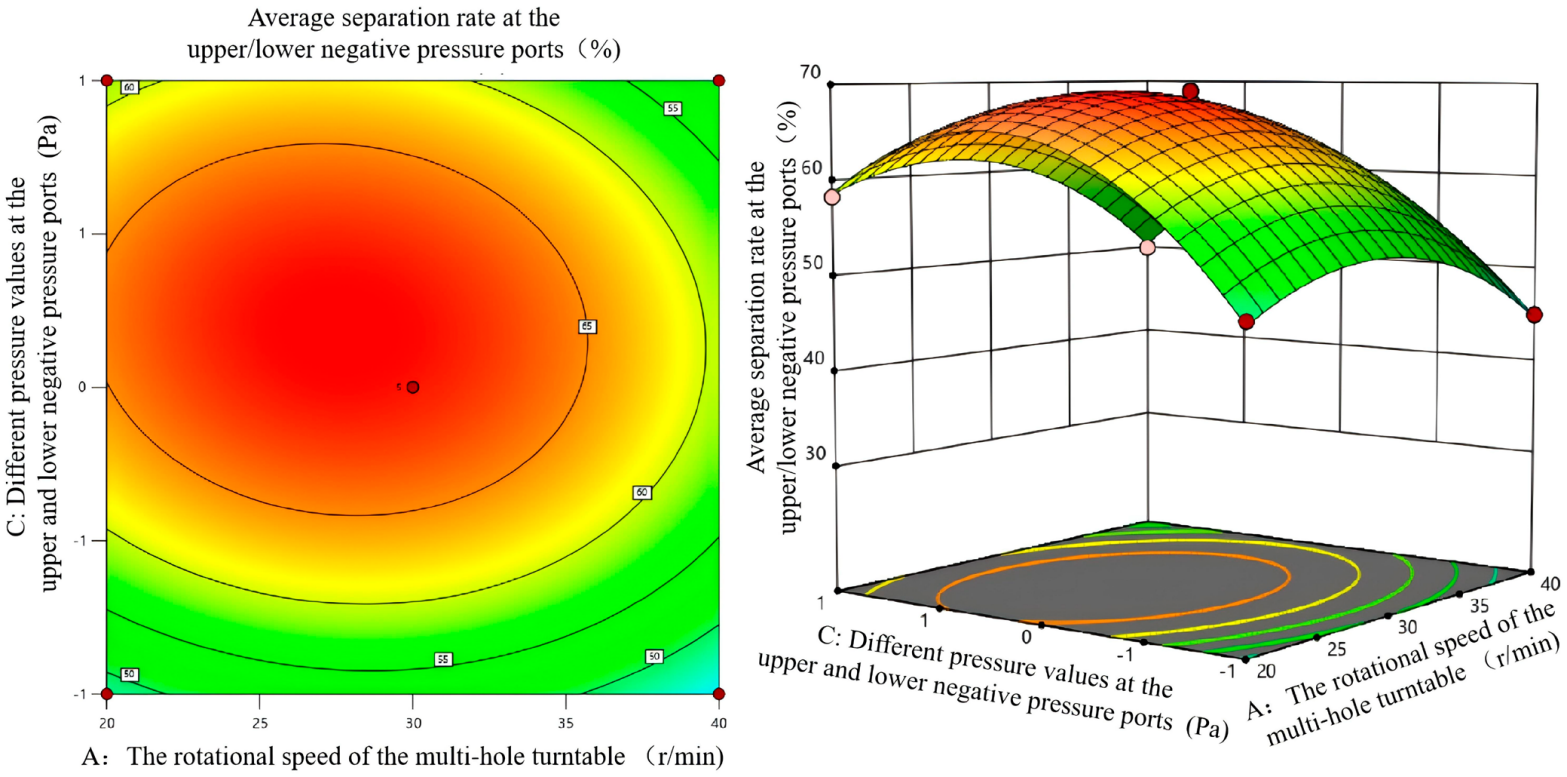
Contour map and response surface map of the influence of the interaction between factors *A* and *C* on the average separation rate under double negative pressure.

**FIGURE 10:**
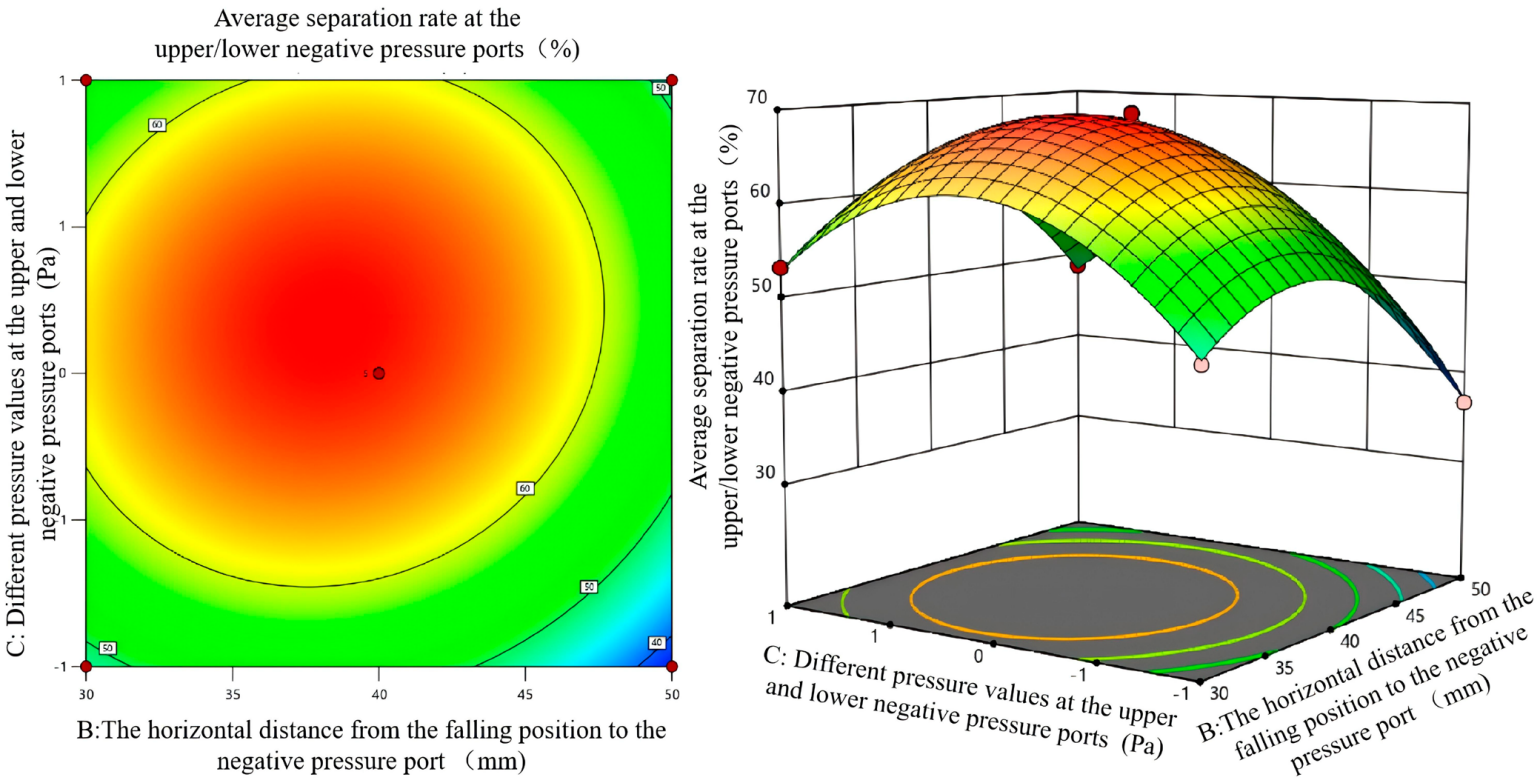
Contour map and response surface map of the influence of the interaction between factors *B* and *C* on the average separation rate under double negative pressure.

According to Table 6, the rotational speed of the multi‐hole turntable, the horizontal distance from the falling position to the negative pressure air outlet, and different pressure values of the upper/lower negative pressure ports have an interactive influence on the success rate of double negative pressure separation. To further analyze the influence of each factor on the success rate of sorting, the response surface diagrams of the influence of each interactive factor on the success rate of sorting were drawn using software, as shown in Figures 8, 9, 10.

Figure 8 shows the influence of the interaction between factors *A* and *B* on the average separation rate under double negative pressure. The contour lines are elliptical, indicating that there is a significant interaction between *A* and *B*; that is, the influence of the two variables on the average separation rate is not independent but interrelated. The average separation rate is the highest in the central red area. The color gradually changes to yellow and green outward, corresponding to a decreasing separation rate. This indicates that the combination of *A* and *B* closer to the central area can achieve a higher average separation rate. In the response surface graph, the steep area of the surface indicates that the variable change has a significant impact on the response value. The surface presents obvious peaks, indicating that there exists an optimal combination of *A* and *B*, which can make the average separation rate reach the maximum value. When *A* is low, as *B* increases, the average separation first rises and then decreases. When *A* is relatively high, a similar trend is also presented, but the peak position varies due to the change of *A*, reflecting the synergistic influence of the two variables on the response value. It can be seen from the contour map and the response surface map that when factor *A* is 30 r/min and factor *B* is 35 mm, the vertex projection of the response surface map is consistent with the center of the contour map, which is the most combined.

Figure 9 shows the influence of the interaction between factors *A* and *C* on the average separation rate under double negative pressure. Among them, the density and shape of the contour lines reflect the intensity of the interaction between the factors. The contour lines in the figure are elliptical, indicating that there is a significant interaction between *A* and *C*. The color changes from dark (red) to light (green) from the center outward, corresponding to a gradual decrease in the average separation rate. The central area is the area with a high separation rate. The morphology of the response surface reflects the nonlinear relationship between the average separation rate and factors *A* and *C*. There is an obvious peak area on the surface, indicating that under a specific combination of *A* and *C*, the average separation rate reaches the maximum value. As *A* or *C* deviates from this peak area, the average separation rate gradually decreases, suggesting that there is a synergistic effect of the influence of the two factors on the separation rate. By observing the densest and darkest area at the center of the contour map, combined with the peak position of the response surface map, the optimal combination is obtained as follows: The rotational speed A is 30–35 r/min, and the pressure value of the negative pressure port is 380/380 Pa.

Figure 10 shows the influence of the interaction between factors *B* and *C* on the average separation rate under double negative pressure. Among them, the depth of the contour line color reflects the level of the separation rate. The reddier the color (in the central area), the higher the separation rate. The contour lines are elliptical, further confirming the interaction between *B* and *C*. Away from the central area, the color becomes lighter (green) and the separation rate gradually decreases. In the response surface graph, the surface color gradually changes from red to green. The red area represents a high separation rate, and the green area represents a low separation rate. The surface shows a trend of first increasing and then decreasing, indicating that under a specific combination of *B* and *C*, the average separation rate reaches the maximum value, and there is a significant interaction between *B* and *C*, jointly affecting the separation rate. Observe the area with the darkest color (red) at the center of the contour map. Combined with the peak position of the response surface map, when the value of factor *B* is 40 mm and the value of factor *C* is 380/380 Pa, the average sorting rate reaches the highest. This combination is the optimal factor interaction combination.

### 4.3 Parameter Optimization and Experimental Verification

According to the analysis of the influence of interaction factors on the test indicators in the previous section, with the average sorting rate of double negative pressure as the goal, the optimal parameters were obtained by optimizing each factor. The rotational speed of the multi‐hole turntable is 30 r/min. The horizontal distance from the falling point to the negative pressure port is 35 mm. The pressure values of the upper and lower negative pressure ports are 380/380 Pa respectively. Similarly, 30 pieces including single leaf and one bud with one leaf, and 20 pieces including one bud with two leaves and one bud with multiple leaves were selected to conduct experiments on the above data. The test results showed that a total of 24 blades were adsorbed at the upper negative pressure port and 15 blades were adsorbed at the lower negative pressure port. The average separation rate of the double negative pressure was 77.5%, and the separation rates were all higher than those of each group of experiments mentioned above.

In conclusion, the double negative pressure separation rates have not yet reached over 90%. The main reasons are as follows: the air pressure at the negative pressure ports and inside the negative pressure chambers is relatively low, resulting in incomplete adsorption by the blades. The position of the negative pressure port and the different angles between the upper and lower negative pressure ports may have better positioning. The vertical distance from the initial position of the fresh tea leaves falling to the negative pressure port also has certain influencing factors on the sorting. The research group will explore the above factors in the subsequent study and compare them with this paper, so as to obtain the combination of factors with a high sorting rate from all angles.

## 5 Conclusion

This paper designs a double negative pressure air suction type tea fresh leaf sorting equipment. The key components are formed by 3D printing. The sorting is achieved by utilizing the adsorption force of the double negative pressure fan and the speed control of the multi‐hole turntable. By using CFD simulation technology, it is concluded that when both the upper and lower negative pressure ports are 380 Pa, the velocity gradient of the flow field is uniform, which is suitable for the sorting of fresh tea leaves at multiple scales. The single‐factor test shows that the separation effect is better when the rotational speed of the multi‐hole turntable is 30 r/min, the horizontal distance between the blade drop and the negative pressure port is 40 mm, and the negative pressure values of both the upper and lower negative pressure ports are 380 Pa. The response surface method was adopted for optimization. The optimal parameters were determined as the rotational speed of the turntable at 30 r/min, the horizontal falling distance at 35 mm, and both the upper and lower negative pressure ports at 380 Pa. The score selection rate of the verification test was 77.5%. Subsequently, the orientation of the negative pressure ports, the spacing of the negative pressure ports, and the distance from the negative pressure ports to the initial position where the fresh tea leaves fall will be considered for research, in order to achieve a high sorting rate at all angles and provide support for the engineering application of efficient sorting equipment for fresh tea leaves.

## Author Contributions

**Xu Zhang:** software (equal), writing – original draft (lead), writing – review and editing (lead), resources (equal), visualization (equal). **Rongyang Wang:** software (equal), resources (equal), visualization (equal).

## Conflicts of Interest

The authors declare no conflicts of interest.

## Data Availability

Data will be provided availability on request.
